# Heart insufficiency after combination of verapamil and metoprolol: A fatal case report and literature review

**DOI:** 10.1002/ccr3.2393

**Published:** 2019-09-19

**Authors:** Eva A. Saedder, Asser Hedegård Thomsen, Jørgen Bo Hasselstrøm, Jakob Ross Jornil

**Affiliations:** ^1^ Department of Clinical Pharmacology Aarhus University Hospital and Aarhus University Aarhus C Denmark; ^2^ Department of Forensic Medicine Aarhus University Aarhus N Denmark

**Keywords:** cardiac insufficiency, cardiovascular toxicology, CYP2D6, drug‐drug interaction, metoprolol, verapamil

## Abstract

The combination of verapamil or diltiazem with beta‐blockers should be avoided because of potentially profound adverse effects on AV (atrioventricular) nodal conduction, heart rate, or cardiac contractility. This effect is unpredictable but may be enhanced due to CYP2D6 poor metabolizer status which could be a special vulnerability factor.

## Abstract 2

The combination of verapamil or diltiazem with beta‐blockers should be avoided because of potentially profound adverse effects on AV nodal conduction, heart rate, or cardiac contractility. This effect is unpredictable but may be enhanced due to CYP2D6 poor metabolizer status which could be a special vulnerability factor.

## Graphical abstract

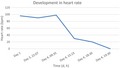

## 1 INTRODUCTION

In selected patients, the combination of nondihydropyrimidine calcium channel blockers with beta‐blockers might provide an effect superior to either drug alone; however, serious and sometimes fatal additive cardiovascular effects occur. This report indicates that CYP2D6 PM status could be a special vulnerability factor for the combination of verapamil and metoprolol.

Calcium channel blockers (CCB) are prescribed for the treatment of arrhythmia and hypertension. Verapamil is a class IV antidysrhythmic drug, which acts by blocking voltage‐sensitive calcium channels. Verapamil is rapidly absorbed and undergoes extensive first‐pass degradation (10%‐20% bioavailability), primarily via O‐ and N‐dealkylation by hepatic cytochrome P450 (CYP) 3A4 forming norverapamil, a pharmacologically active metabolite of verapamil. Verapamil and its metabolite have the ability to inhibit transmembrane calcium flux in cardiac cells and smooth muscle cells. Its pharmacological effects are reduction in heart rate and myocardial contractility, slow atrioventricular (A–V) node conduction, and reduction in the peripheral vascular resistance.^1^ Verapamil inhibit CYP3A4 and P‐glycoprotein–mediated drug transport, which may alter the intestinal absorption of several drugs and their distribution into peripheral tissues and the central nervous system.^1^ During overdose, half‐life of verapamil may be greatly prolonged (up to 10 days); this may be due to saturation of the hepatic enzyme or rate‐limiting absorption.^2^

Beta‐adrenergic blockers (BB) are used in the treatment of hypertension and heart failure. Metoprolol is a selective b1‐adrenergic blocking agent, and it is lipophilic and predominately metabolized in the liver via cytochrome CYP2D6. Blockade of the myocardial b1 receptor reduces heart rate, myocardial contractility, and cardiac output.^3^ Dizziness, bradycardia, and hypotension are observed as adverse reactions at therapeutic plasma levels.

Studies from the 1980s suggested that in selected patients, the combination of nondihydropyrimidine CCBs like verapamil with BBs like metoprolol might provide an effect superior to either drug alone; however, many studies and case reports have provided data that confirm serious and sometimes fatal additive cardiovascular effects. Here we report a fatal case of heart insufficiency after the combination of verapamil and metoprolol and supply with an overview of the available literature.

## 2 CASE REPORT

A 76‐year‐old woman who was suffering from persistent atrial fibrillation, atrial hypertension, and chronic obstructive lung disease and who had previously been in treatment for ovarian cancer, colon cancer, and breast cancer was hospitalized due to an INR (international normalized ratio) above 9.0. At the time of hospitalization, she was in treatment with verapamil 120 mg daily and warfarin for persistent atrial fibrillation. A complete list of medicine at the time of hospitalization is available in Table 1.

**Table 1:** List of medicine at the time of hospitalization

Drug	Dose	Times daily	Indication
Verapamil	120 mg	1	Atrial fibrillation
Warfarin			Atrial fibrillation
Pantoprazole	40 mg	1	Heartburn
Potassium	40 mL	1	Hypokalaemia
Losartan/Thiazide	100 + 25	1	Atrial hypertension
Furosemide	40 mg	1	
Pregabalin	75 mg	2	
Zopiclone	7.5 mg	Prn, max × 1	Insomnia
Salbutamol	0.2 mg	prn	COPD
Fluticasone/Salmeterol	50 + 500 µg	2	COPD
Tiotropium	5 mg	1	COPD
Povidone			
Paracetamol	1000 mg	4	Pain
Fluconazole	100 mg	1	Fungal infection
Tramadol	50 mg	3	Pain
Penicillin	1.5 mi.e		Cystitis

The patient had recently had a gastroscopy revealing a fungal infection and a high level of gastric acid. Her family physician therefore initiated a treatment with a short course of fluconazole and pantoprazole. After 2 days of treatment with fluconazole, her physician measured an INR of 5.6. After a control visit 2 days later, the INR had increased to eight and the patient was hospitalized.

**Figure 1:**
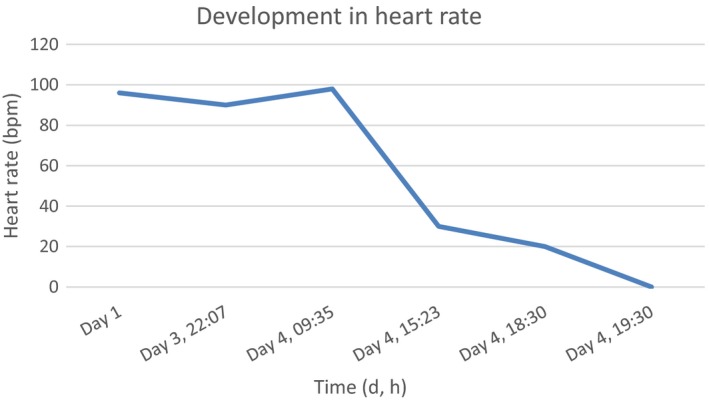
The development in heart rate from day 1 to day 4, where the patient dies

At the time of hospitalization, her heart rate was 96 bpm. During the evening on the third day of hospitalization, an electrocardiogram (ECG) showed atrial fibrillation and a junior physician prescribed Selo‐zok^®^ (metoprolol), 50 mg slow‐release tablet. According to the latest guideline from the European Society of Cardiology, a patient in need of acute rhythm control can have digoxin added to the treatment with verapamil, if the patient has a left ventricular ejection fraction of above 40 and the heart rate is above 110 bpm.^4^ The patient was not known with previous heart failure or reduced ventricular ejection fraction, and a suspicion of heart failure was not mentioned in the hospital records at this time. The next morning an experienced doctor discontinued metoprolol during the morning rounds, as she was aware of a potential interaction between metoprolol and verapamil. The patient was well and had no signs of acute illnesses. Only one tablet of metoprolol 50 mg had been administered to the patient. After lunch on the same day, the patient developed bradycardia and hypotension and infusion with isoprenaline was initiated (see Table 2 for details). An interaction between verapamil and metoprolol was suspected. The condition progressed and despite of isoprenaline, atropine, and external pacing, her blood pressure was immeasurable and her heart rate decreased (Figure 1).

**Table 2:** A time schedule of events

Day	Time	Event
1	22:07	Prescribed metoprolol 50 mg
2	09:35	Discontinued metoprolol after only 1 dose of 50 mg given on the night before
	15:23	Hypotension and low pulse (frequency of 30)
		Isoprenaline infusion 20‐60 mL/h
	15:36	Intensive care due to cyanosis and no measureable pulse
	17:22	Isoprenaline infusion 60 mL/h. Pulse 30. Decreased consciousness. No effect of atropine.
	18:15	Intubation and mechanical respiration. Hypotensive, systolic blood pressure 90. pH 7.1. Transfer to other hospital planned.
	18:30	During transportation: unconscious, cold, frequence on scope 20‐25, no palpable pulse, severely reduced ejection fraction, some effect of adrenalin 50 µg refracted doses, external pacing.
	18:45	Arrived at other hospital. Cold and cyanosis. Dilated pupils, infusion of dopamine 10 µg/kg/min. Adrenaline. No response on heart function.
	19:30	The patient dies.

She was transferred to another hospital for the insertion of a temporary pace wire. On arrival at the second hospital, the patient was completely unresponsive, she had been intubated in the ambulance and her pulse had decreased to ten beats per minute. Blood analysis showed serious metabolic acidosis. Further treatment was considered futile, and the patient was declared dead at 19.30 on the third day of hospitalization. A medicolegal autopsy was performed, and a standard forensic toxicological analysis was performed on blood withdrawn from the femoral vein at autopsy shortly after her death, see Table 3 for the blood concentrations of drugs. The autopsy showed right atrial dilatation, but otherwise normal right and left ventricles, normal heart valves, and no signs of acute coronary syndrome, and the liver was normal.

**Table 3:** Concentration of drugs found in postmortem femoral blood

Drug	Concentration
Atropine	0.036 mg/kg
Fentanyl	0.00080 mg/kg
Fluconazole	9.1 mg/kg
Furosemide	1.3 mg/kg
Ketamine	0.060 mg/kg
Lidocaine	0.011 mg/kg
Losartan	0.032 mg/kg
Metoprolol	0.50 mg/kg
Midazolam	0.0082 mg/kg
Morphine	0.13 mg/kg
Oxycodone	0.10 mg/kg
Paracetamol (acetaminophen)	26 mg/kg
Pregabalin	3.7 mg/kg
Salbutamol	0.0011 mg/kg
Tramadol	0.038 mg/kg
Tramadol, O‐desmethyl	0.0025 mg/kg
Tramadol, N‐desmethyl	0.14 mg/kg
Verapamil	0.24 mg/kg
Warfarin	0.15 mg/kg
Zopiclone	0.062 mg/kg

## 3 DISCUSSION

Most importantly, the toxicological analysis revealed a whole blood concentration of metoprolol of 0.50 mg/kg and verapamil of 0.24 mg/kg.

Morphine, oxycodone, and fentanyl were not assessed to be of importance due to supportive treatment with respirator. Other drugs were found at levels normally seen in treatment or below.

Known therapeutic whole blood concentration ranges of verapamil are 0.015‐0.19 mg/kg and of metoprolol are 0.039‐0.55 mg/kg 5 (recalculated from plasma values using blood/plasma ratio 1.1 for metoprolol and 0.75 for verapamil 6). Known metoprolol whole blood concentrations from drug‐induced fatalities average 60 mg/kg (range 4.7‐142) and verapamil concentrations average 11 mg/kg (range 0.9‐85).^6^ In this case, one single tablet of metoprolol 50 mg was administered, and the blood concentration of metoprolol was found to be in the high end of the therapeutic concentration range almost 24 hours later, despite of an elimination half‐life of metoprolol slow‐release tablets of 3‐4 hours. Postmortem redistribution might have caused an increase in concentrations; however, the patient died from a serious cardiac insufficiency, which points in the direction of an interaction between metoprolol and verapamil.

The efficacy and safety data supporting the use of CCBs and BBs primarily comes from monotherapy, and clinical studies on the combined use mainly concern the treatment of angina pectoris in patients with chronic coronary heart disease.^7^, ^8^, ^9^ Worsening of myocardial function, such as hypotension, bradycardia, and AV block, might be expected to occur more often with combination therapy rather than therapy with either drug alone.^7^, ^10^ Some authors found that cardiac risk increases by left ventricular dysfunction, aortic stenosis, low‐pulse rate, or large doses of either drug 10, 11, 12, 13, 14; however, other authors describe cases in which the ventricular function was normal or near normal and incidents have often occurred at normal doses of both drugs (Table 4).^15^

**Table 4:** Published case reports

Reference	Age (y)	Gender	Dose (mg/d)	Serum/blood level (mg/kg)	Symptoms	Treatment
Mills TA 2004^35^	61	F	Verapamil 360 Propranolol 40		Sinus bradycardi (26/min) Junctional escape rhythm	Cessation of treatment
	58	F	Atenolol 100 Diltiazem 360		Hypotension (87/45) Atrial bradycardia (12/min) Junctional escape rhythm	Atropine, temporary pacing
	62	F	Diltiazem 240 Enalapril		Sinus bradycardia (31/min) Junctional escape rhythm	Cessation of treatment
	73	F	Diltiazem 120 Atenolol 25		Sinus arrest Junctional escape rhythm	Atropine, dopamine, external pacing
	73	F	Metoprolol 50 Diltiazem 180		Sinus bradycardia (34/min) Junctional escape rhythm	Stopped treatment
	61	M	Nadolol 40 Diltiazem 300		Sinus arrest Junctional escape rhythm	Atropine, temporary pacemaker
	62	M	Verapamil 360 Atenolol 25		Sinus bradycardia (54/min)	Cessation of treatment Complicated by chronic kidney disease and hemodialysis
	73	F	Verapamil 480 Metoprolol 200		Hypotension (98/64) Sinus bradycardia (39/min) Junctional escape rhythm	Cessation of treatment
	60	M	Metoprolol 150 Amlodipine 20		No symptoms, sinus pause revealed by holter	Metoprolol stopped
Sakurai H 2000^24^	54	M	Verapamil 360 Metoprolol 200		Shock, Pulmonary edema, bradycardia (56/min) Junctional escape rhythm	Dopamine, furosemide
	69	F	Verapamil 240 Metoprolol 100		Shock, pulmonary congestion, sinus bradycardia (44/min)	Isoprenaline
	60	F	Verapamil 160 Pindolol 10		Hypotension, sinus bradycardia (40/min)	Cessation of treatment
	53	M	Verapamil 480 Propranolol 160		Hypotension, bradycardia (32/min), AV nodal rhythm	Isoproterenol, dopamine
	55	F	Verapamil 80 Propranolol 80		Hypotension, bradycardia	Epinephrine
	21	F	Verapamil NA Atenolol NA	0.367 0.65	Shock, bradycardia, AV nodal rhythm	Calcium chloride
	42	M	Verapamil 120 Atenolol 50		Shock, sinus arrest	Dopamine, temporary pacing
	57	F	Verapamil NA Atenolol NA	0.45 1.7	Shock, complete heart block	Dopamine, dobutamine, noradrenaline, temporary pacing, intraaortic balloon
	78	F	Verapamil 240 Metoprolol 100		Shock, complete heart block	Calcium gluconate
	72	F	Verapamil 160 Atenolol 50		Shock, pulmonary congestion, electromechanical dissociation	Calcium chloride
Robson RH 1982^36^	60	M	Nifedipine 60 Atenolol 100		Congestive heart failure	Cessation of treatment
Staffurth JS 1981^20^	47	M	Nifedipine 30 Propranolol 640		Hypotension (unrecordable), pulse rate 48/min	Cessation of treatment
Eisenberg JNH 1984^23^	46	M	Verapamil 240 Metoprolol 200		Bradycardia (44/min), Wenckebach AV block	Cessation of verapamil
Anastassiades CJ 1980^22^	72	M	Nifedipine 400 Alprenolol 30		Dyspnoea, pulmonary edema	Cessation of nifedipine
	58	M	Nifedipine 30 Propranolol 120		Dyspnoea, edema of the legs, congestive heart failure	Cessation of treatment

That the combined use of CCBs and BBs may cause adverse cardiovascular effects was seen in the clinical trials investigating combined use, and it has been clinically documented (Table 4).^7^, ^12^, ^15^, ^16^, ^17^, ^18^, ^19^, ^20^, ^21^, ^22^, ^23^, ^24^ The precise nature of the mechanism is uncertain, and it may be due to the combination of a number of actions, both pharmacokinetic and pharmacodynamic.

An existing interaction between metoprolol and verapamil is well documented. Verapamil has been shown to affect the clearance of the lipophilic BBs, propranolol, and metoprolol (both metabolized in the liver), but to have no effect on the pharmacokinetics of atenolol, a hydrophilic compound excreted unchanged in the urine.^11^, ^25^, ^26^, ^27^ McCourty et al investigated the effect of verapamil on the pharmacokinetics and pharmacodynamics of propranolol in six patients and found an increase in the area under the curve (AUC) of propranolol, that however, did not reach statistical significance.^11^ The six patients received the same doses, but AUC of propranolol differed statistically significantly between the subjects. One patient was withdrawn from the study as his ECG showed atrioventricular dissociation with a ventricular rate of 37 bpm. His AUC is not presented in the paper. Concomitant administration of metoprolol with verapamil produced a significant increase in peak plasma concentration and in the AUC of metoprolol by 85%, respectively 35%.^25^, ^26^ Keech et al investigated the pharmacokinetic interaction between metoprolol and verapamil in nine patients.^26^ One patient collapsed with profound sinus bradycardia and hypotension.

The inhibitory effects of six CCBs, including verapamil and diltiazem, on three major CYP isoenzymes, CYP2C9, CYP2D6, and CYP3A4, were examined in liver microsomes.^27^ All six compounds reversibly inhibited CYP2D6, CYP2C9, and with increasing potency, CYP3A4.

Four metabolizer phenotypes characterize drug metabolism via CYP2D6 in vivo: ultrarapid metabolizer (UM), extensive metabolizer (EM), intermediate metabolizer (IM), and poor metabolizer (PM).^28^ Based on the genotype involved, the plasma concentration of metoprolol may range from subtherapeutic levels in the UM group to supratherapeutic and potentially toxic concentrations in the PM group, increasing the probability of adverse effects such as hypotension and bradycardia.^29^ A systematic review from 2013 found differences in peak plasma metoprolol concentration, AUC, elimination half‐life, and apparent oral clearance that were 2.3‐, 4.9‐, 2.3‐, and 5.9‐fold between EM and PM, respectively and 5.3‐, 13‐, 2.6‐, and 15‐fold between UM and PM (all *P* < .001), respectively.^30^

The ratio between tramadol (TRA) and the metabolite O‐desmethyltramadol (ODT) can for living individuals be used to estimate an individual's CYP2D6 phenotype.^31^ In a postmortem setting, the ratio has been used to estimate an individual's CYP2D6 genotype.^32^, ^33^ A TRA/ODT ratio above 15‐30 indicates CYP2D6 PM genotype. In this case, the TRA/ODT ratio was 15 indicating CYP2D6 PM genotype. The ratio TRA/ODT is not that specific in predicting CYP2D6 PM phenotype, but Fonseca et al found that a ratio between N‐desmethyltramadol (NDT) and ODT above seven was a more predictive ratio for CYP2D6 PM genotype.^33^ In this case, NDT/ODT = 56 gives a strong indication of our patient being CYP2D6 PM genotype.

Another possible interaction caused by verapamil could occur via inhibition of organic cation transporter OCT1 which would cause a reduced uptake of metoprolol into the hepatocytes, and thus, a decrease in metabolism.^34^ This means that individuals with CYP2D6 PM status who receive a combination of verapamil and metoprolol would be especially in risk of attaining high metoprolol concentrations and also have a slow elimination of metoprolol.

In the above case, a junior MD ordered one single tablet of metoprolol 50 mg, and for some reason, the blood concentration of metoprolol was much higher than expected almost 24 hours later. Even though postmortem redistribution might have caused an increase in concentrations, the patient died from a serious cardiac insufficiency, which points in the direction of an interaction between metoprolol and verapamil. This interaction might have been strong due to CYP2D6 PM status which could be a special vulnerability factor for the combination of verapamil and metoprolol.

## CONFLICT OF INTEREST

None declared.

## AUTHORS CONTRIBUTIONS

Eva A. Sædder: was responsible for writing the manuscript and for medical interpretation of blood samples and toxicology (first author). Asser Hedegård Thomsen: was the responsible forensic pathologist and performed the autopsy. Jørgen Bo Hasselstrøm: was responsible for the laboratory tests performed. Jakob Ross Jornil: was the responsible analytical chemist in the case. All authors: read, contributed to, and approved the final manuscript.
